# Association of GSTM1 Null Allele with Prostate Cancer Risk: Evidence from 36 Case-Control Studies

**DOI:** 10.1371/journal.pone.0046982

**Published:** 2012-10-10

**Authors:** Bingbing Wei, Zhuoqun Xu, You Zhou, Jun Ruan, Huan Cheng, Bo Xi, Ming Zhu, Ke Jin, Deqi Zhou, Qiang Hu, Qiang Wang, Zhirong Wang, Zhiqiang Yan, Feng Xuan, Xing Huang, Jian Zhang, Hongyi Zhou

**Affiliations:** 1 Department of Urology, Affiliated Wuxi People’s Hospital, Nanjing Medical University, Wuxi, China; 2 Faculty of Medicine, University of Helsinki, Finland; 3 Department of Urology, First Affiliated Hospital of Nanjing Medical University, Nanjing, China; 4 Department of Maternal and Child Health Care, School of Public Health, Shandong University, Jinan, China; The University of Texas M. D. Anderson Cancer Center, United States of America

## Abstract

**Background:**

Glutathione S-transferase M1 (GSTM1) is thought to be involved in detoxifying several carcinogens and may play a vital role in tumorigenesis. Numerous studies have evaluated the association between GSTM1 null/present polymorphism and risk of prostate cancer (PCa). However, the results remain inconsistent. To derive a more precise estimation, we performed a meta-analysis.

**Methodology/Principal Findings:**

A comprehensive search was conducted to identify all eligible case-control studies. We used odds ratios (ORs) with 95% confidence intervals (CIs) to assess the strength of the association. The overall association was significant (OR = 1.28, 95% CI: 1.11–1.48, *P* = 0.001). Moreover, subgroup analyses showed GSTM1 null genotype significantly associated with PCa risk among Asians (OR = 1.35, 95% CI: 1.03–1.78, *P* = 0.03) but not among Caucasians (OR = 1.12, 95% CI: 0.96–1.31, *P* = 0.16). In addition, we did not find that smoking modified the genotype effect on the risk of PCa.

**Conclusions/Significance:**

The present meta-analysis suggested that GSTM1 null allele was a low-penetrant risk factor for PCa among Asians.

## Introduction

Prostate cancer (PCa) is now thought to be one of the most important medical problems in the male population [1]. In European countries, it is recognized as the most common solid neoplasm, with an incidence rate of 214 cases per 1000 men, outnumbering lung and colorectal cancer [2]. Furthermore, PCa is currently the second most common cause of cancer death in men [3]. Genetic predisposition and environmental factors are likely to contribute to the risk of PCa [4]; however, the etiology of PCa remains unclear. PCa incidence rate varies remarkably in different populations, highest among Africans, intermediate among Caucasians and lowest among Asians [5]. The variation in different ethnicities suggests that the genetic and environmental factor may play an important role in the etiology of PCa.

Generally, genetic susceptibility could modify the effect of environmental exposure, possibly explaining the difference of PCa incidence rate throughout the world. It is possible that the susceptibility to PCa is determined by the interindividual differences in the bioactivation of procarcinogens and detoxification of carcinogens because of the polymorphisms in metabolic genes. Glutathione S-transferase M1 (GSTM1) is thought to be involved in detoxification of carcinogens, which has been considered as a PCa susceptibility gene [6].

GSTM1, located on chromosome 1p13.3, detoxifies numerous electrophilic substances, including carcinogens such as polycyclic aromatic hydrocarbons, ethylene oxide, epoxides, and styrene. GSTM1 expression could be hormonally controlled and induced by phenobarbital or propythiouracil [7]. Three genetic polymorphisms, namely GSTM1*0 (GSTM1 null polymorphism), GSTM1*A and GSTM1*B, have been identified. GSTM1*0 is a deleted allele, and the homozygous allele (GSTM1 null genotype) is thought to be associated with low ability to detoxify several xenobiotics, reduced defense ability against oxidative stress, and free radical-mediated cellular damage [8]–[10]. Many studies on GSTM1 null genotype and PCa have compared the homozygous deletion genotype with the genotypes containing at least one functional allele (Null versus Present) [11]–[44]. Because GSTM1 null genotype could affect PCa risk by mediating the detoxification of activated tobacco carcinogens, it is with great interest that the tobacco smoking might affect the association between GSTM1 null genotype and PCa risk. Recent years, several studies have evaluated this possible effect [11]–[44]. However, the results are contradictory because of relatively small sample size with low statistical power. We therefore conducted a meta-analysis in order to provide an accurate estimate of the association.

**Figure 1:**
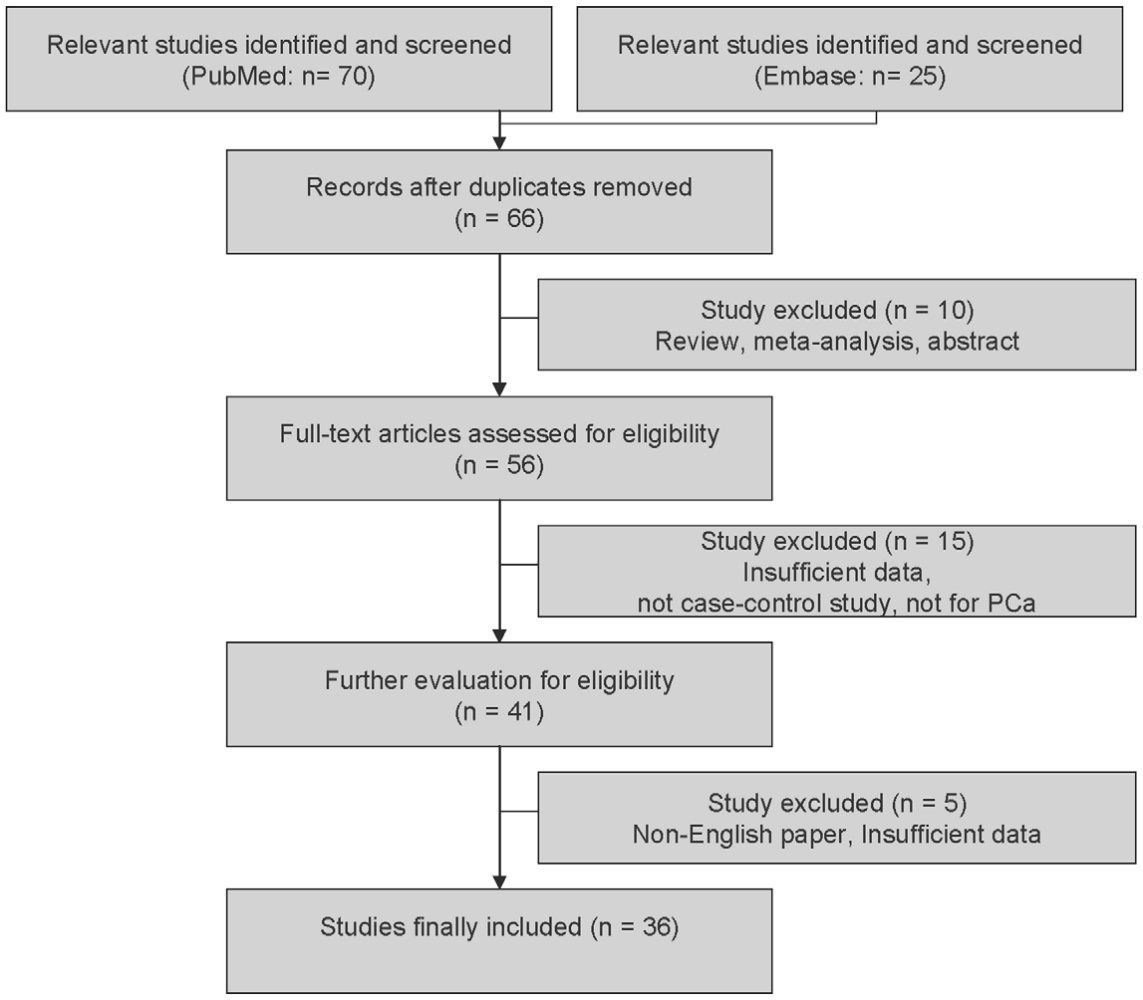
Flow chart of study selection based on the inclusion and exclusion criteria.

## Methods

### Identification and Eligibility of Studies

PubMed (1956 to July 2012) and Embase (1947 to July 2012) database search was conducted using the following search terms: “GSTM1 or GST”, “polymorphism or variant”, and “prostate or prostatic”. Additional relevant studies were identified by a hand search of the references of original studies. Of these studies with the same or overlapping data, we selected the most recent ones with the largest number of subjects. Studies included in this meta-analysis should meet the following criteria: (a) evaluation the association of GSTM null/present polymorphism and PCa risk published in English language, (b) case-control study, (c) containing sufficient data for estimation of odds ratio (OR) with 95% confidence interval (95% CI).

**Figure 2:**
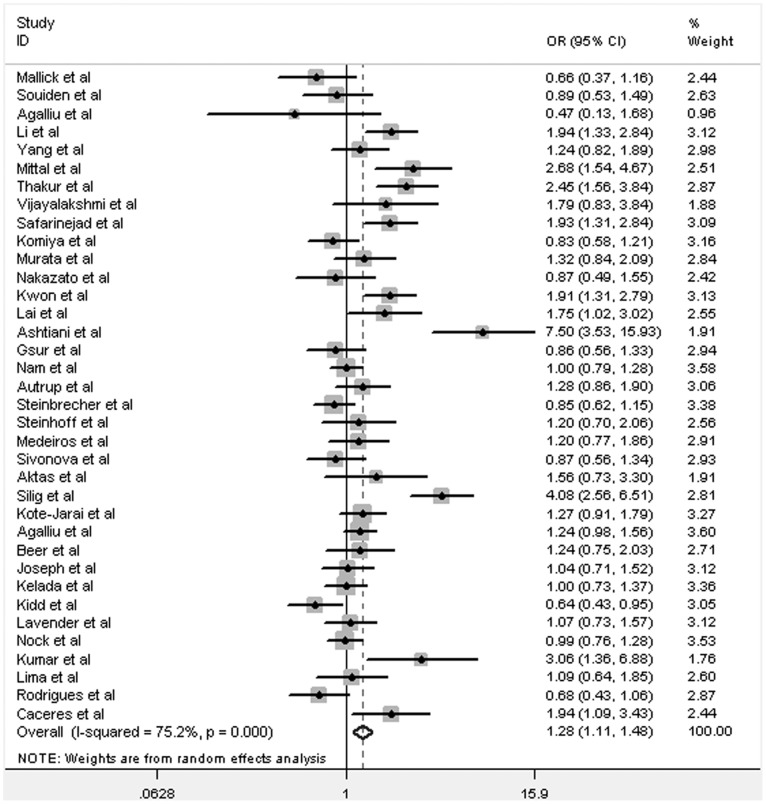
Forest plot of PCa risk associated with GSTM1 null polymorphism (for Null versus Present). The squares and horizontal lines correspond to the study-specific OR and 95% CI. The area of the squares reflects the weight (inverse of the variance). The diamonds represent the summary OR and 95% CI.

### Data Extraction

Two authors independently extracted the data and reached a consensus on all the items. For each study, the following information was collected: first author, publishing year, ethnicity of subjects, source of controls, number of cases and controls, genotyping method. Different ethnic descents were categorized as Caucasians, Asians, and Africans. If a study did not specify the ethnicity or if it was not possible to separate participants according to such phenotype, the group was termed “mixed ethnicity”. For study [21] including subjects of different ethnic populations, data were collected separately whenever possible and recognized as an independent study.

**Figure 3:**
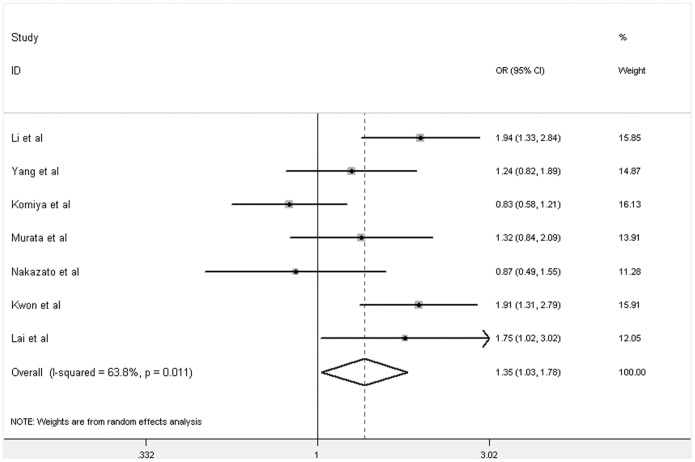
Forest plot of PCa risk associated with GSTM1 null polymorphism among Asians (for Null versus Present). The squares and horizontal lines correspond to the study-specific OR and 95% CI. The area of the squares reflects the weight (inverse of the variance). The diamonds represent the summary OR and 95% CI.

**Figure 4:**
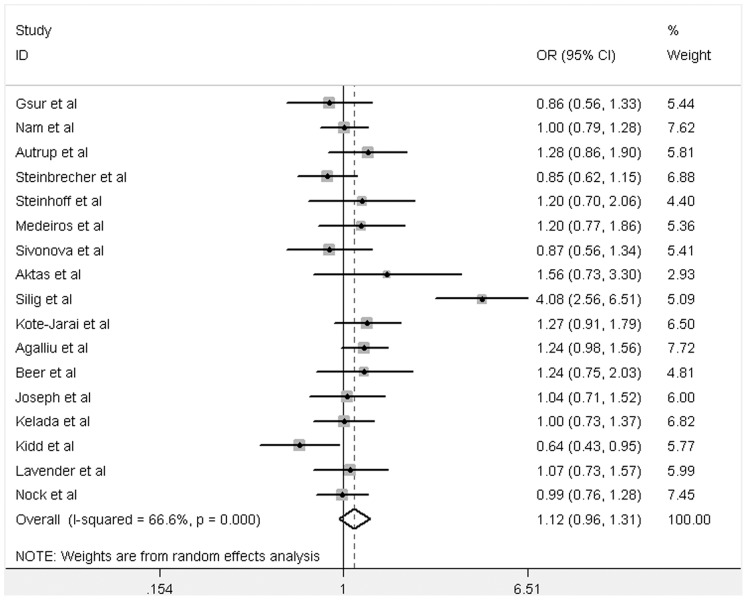
Forest plot of PCa risk associated with GSTM1 null polymorphism among Caucasians (for Null versus Present). The squares and horizontal lines correspond to the study-specific OR and 95% CI. The area of the squares reflects the weight (inverse of the variance). The diamonds represent the summary OR and 95% CI.

### Statistical Analysis

The strength of the association between GSTM1 null/present polymorphism and PCa risk was measured by ORs with 95% CIs. The statistical significance of the summary OR was determined by the Z-test. For GSTM1 null polymorphism, we estimated the risk of the “Null” genotype on PCa risk, compared with the “Present” genotype. Stratified analyses were performed by ethnicities and smoking status.

**Figure 5:**
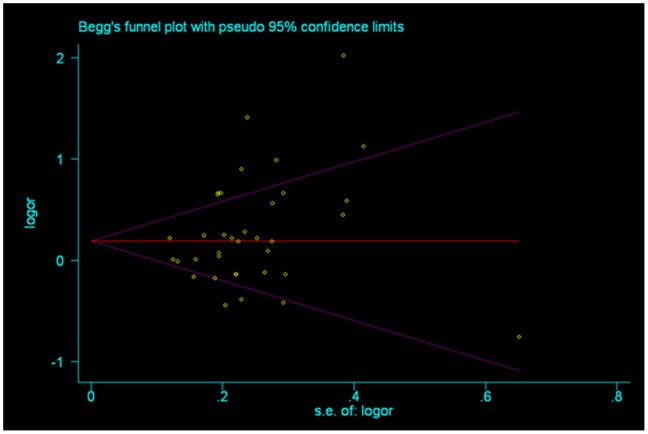
Begg’s funnel plot for publication bias test [for Null versus Present]. Each point represents a separate study for the indicated association. Log[or], natural logarithm of OR. Horizontal line, mean effect size.

Heterogeneity was evaluated by χ^2^-based Q-test. A *P* value of greater than 0.10 indicates a lack of heterogeneity among studies and the fixed-effects model was used to estimate the pooled OR of each study (the Mantel-Haenszel method). Otherwise, the random-effects model (the DerSimonian and Laird method) was used [45], [46]. Sensitivity analysis was performed to assess the stability of results. Begg’s funnel plot and Egger’s test were performed to assess the publication bias of literatures; *P*<0.05 was considered statistically significant.

All statistical tests for this meta-analysis were performed with STATA (version 10.0; Stata Corporation, College Station, TX).

## Results

### Eligible Studies

For PCa risk related to GSTM1 null polymorphism, articles were retrieved based on the search criteria above. Study selection process is shown in Figure 1. Ninety-five articles were retrieved. However, there were obvious overlapping data among a number of studies [23], [34], [39], [42], [47]–[51]. According to our inclusion criteria, some of them were included [23], [34], [39], [42], [51]. Finally, a total of 36 studies including 6,202 cases and 8,209 controls were eligible for the meta-analysis. Study characteristics are summarized in Table S1. There were 7 studies on subjects of Asian, 17 of Caucasian, 3 of African and 9 of mixed ethnicity. Among them, 6 studies evaluated the effect of smoking status on the association. The controls from all eligible studies were frequency-matched controls to cases by age and ethnicity. A classical PCR or multiple PCR assay was conducted in 31 studies.

### Meta-analysis

Overall, we found that GSTM1 null genotype was significantly associated with increased risk of PCa (Null versus Present: OR = 1.28, 95% CI: 1.11–1.48, *P* = 0.001; Table S2, Figure 2). In addition, subgroup analyses showed that there was significant association among Asians (Null versus Present: OR = 1.35, 95% CI: 1.03–1.78, *P* = 0.03; Table S2, Figure 3), but neither in Caucasians (Null versus Present: OR = 1.12, 95% CI: 0.96–1.31, *P* = 0.16; Table S2, Figure 4) nor Africans (data not shown).

### GSTM1-smoking Interaction

The data on GSTM1 null genotype stratified by smoking status were available in six studies. Non-smokers with the GSTM1 null genotype did not have a significantly increased PCa risk, compared to Present genotype (Null versus Present: OR = 1.25, 95% CI: 0.64–2.45, *P* = 0.52; Table S2). In addition, there was no significant association between GSTM1 null polymorphism and PCa risk among smokers (Null versus Present: OR = 1.16, 95% CI: 0.95–1.43, *P* = 0.15; Table S2). This result was further confirmed by logistic regression analyses (data not shown).

### Test of Heterogeneity

The heterogeneity was reckoned between each of the studies using Q-test. Overall significant heterogeneity was detected across studies (Null versus Present: *P_heterogeneity_* <0.01; Table S2). In stratified analysis by ethnicity, there was significant heterogeneity among Asians (*P_heterogeneity = _*0.01), Caucasians (*P_heterogeneity_* <0.01; Table S2).

### Sensitivity Analysis

In the sensitivity analysis, the influence of each study on the pooled OR was examined by repeating the meta-analysis while omitting each study, one at a time. This procedure confirmed the stability of our overall result (data not shown).

### Publication Bias

Begg’s funnel plot and Egger’s test were conducted to assess a possible publication bias in the literature. The shapes of funnel plots did not reveal any evidence of funnel plot asymmetry. The results Egger’s test from showed no indication of publication bias (*P* = 0.08; Figure 5).

## Discussion

We performed a systematic literature search and combined the available results in the present meta-analysis, which is a useful strategy for elucidating genetic factors in cancer [52]. GSTM1 is thought to be involved in detoxification of hydrophobic electrophiles or oxidized lipids derived from the metabolism of xenobiotics [53], [54]. The association of GSTM1 null polymorphism with different cancers, such as lung cancer [55], gastric cancer [56], and bladder cancer [57], has been extensively explored. The results of published studies on the association between GSTM1 null polymorphism and PCa risk remain conflicting and contradictory. The inconclusive results may be due to a small effect of GSTM1 null polymorphism on PCa risk. The relatively low statistical power of published studies with small sample size might be a reason as well. Mo et al [58] evaluated the association between GSTM1 null polymorphism and PCa risk by meta-analysis 3 years ago, but their study included overlapping data which might cause the bias. In addition, their use of scales for assessing quality or risk of bias is explicitly discouraged in Cochrane reviews [59]. Moreover, recently many original studies have come out and provided a variety of results. Hence, we conducted an updating meta-analysis to provide a quantitative approach for combining all the available within same topic. Concerning the reason methodology rules of meta-analysis [60], [61], our study might provide a more accurate estimation.

The present meta-analysis, including 6,202 cases and 8,209 controls, explored the association between GSTM1 null/present polymorphism and PCa risk. Overall, we found that GSTM1 null genotype was significantly associated with PCa risk. Moreover, the association remained significant among Asians but not Caucasians. GSTM1 is thought to be involved in detoxification of hydrophobic electrophiles or oxidized lipids derived from the metabolism of xenobiotics [53], [54], and play a vital role in carcinogenesis. The GSTM1 null genotype has no enzymatic activity and may affect the detoxification of carcinogens. Thus, it might be capable to alter the susceptibility to PCa among Asians. Given the important biological roles, the significant association between GSTM1 null polymorphism and PCa risk is reasonable and convincing.

In this study, we reported a significant association between GSTM1 null polymorphism and PCa risk among Asians, but not Caucasians. Ethnicity is a well established confounding factor for PCa. Our result suggested a possible role of ethnic difference in genetic backgrounds and the environment they lived in [62]. Actually, a number of studies have demonstrated that GSTM1 null genotype is significantly associated with some other cancers only in Asians. Zhuo et al [63] found that GSTM1 null genotype significantly increased susceptibility to oral cancer among Asians, but not Caucasians. Wang et al [64] reported that the null genotype of GSTM1 was a risk factor in cervical cancer among Asians, but not Caucasians. A recent study by Wang et al [65] showed that an increased hepatocellular carcinoma risk was significantly affected by the null genotype of GSTM1 among Asians. In addition, a meta-analysis by Qiu et al [66] provided evidences that the GSTM1 null genotype is a low-penetrant risk factor for gastric cancer development only in the Asian population.

To date, there is no reasonable molecular mechanism to explain the result. In the Caucasian population, the effect of the null genotype of GSTM1 on PCa risk might be masked by the presence of other as-yet unidentified causal genes involved in the development of PCa. In addition, heterogeneous exposure patterns to chemicals and environmental risk factors might also be taken account into account for the difference. Because there was relatively small sample size for African population, the result on Africans should be interpreted with caution.

GSTMl is involved in detoxification of epoxides from carcinogenic polycyclic aromatic hydrocarbons and combination of exposure to cigarette smoking. Lack of GSTMl activity would increase the burden with ultimate carcinogenic epoxides [33]. In subgroup analyses by smoking status, the significant association was neither detected among non-smokers nor smokers. This suggested that smoking might not significantly modify the effect of GSTM1 null polymorphism on PCa risk. However, the result on undetected effect should be interpreted with caution because of a relatively small sample size included in the study.

Several limitation of our study should be addressed. (i) Although case misclassification bias was unlikely to exist in this study because all PCa cases were confirmed on the basis of histological criteria, we could not exclude the possibility that some control subjects had latent PCa which had not been detectable by PSA analysis or digital rectal examination (DRE). The undetected PCa in controls might produce bias estimates toward the null, and the strength of positive correlation might be underestimated. (ii) Our result was based on unadjusted estimates because of the limited information available. A more precise analysis should be conducted on the basis of adjustment for confounders such as age. (iii) Data on Africans was limited.

In summary, this meta-analysis provided the evidence that GSTM1 null genotype might be a low-penetrant susceptibility marker for PCa among Asians.

## Supporting Information

Table S1 **The characteristics of studies included in the meta-analysis.**
(DOC)

Table S2 **Main result of pooled ORs in the meta-analysis.**
(DOC)
