# Supplementary material for: Association of GSTM1 Null Allele with Prostate Cancer Risk: Evidence from 36 Case-Control Studies
Source: PLoS One. 2012 Oct 10;7(10):e46982. doi: 10.1371/journal.pone.0046982 (PMC3468624; doi:10.1371/journal.pone.0046982)
Supplement: Table S1 — The characteristics of studies included in the meta-analysis. (DOC) [file pone.0046982.s001.doc]

**Table S1** The characteristics of studies included in the meta-analysis.

| Author | Year | Ethnicity | Cases | | Controls | |
| --- | --- | --- | --- | --- | --- | --- |
|  |  |  | Present | Null | Present | Null |
| Mallick et al | 2007 | African | 108 | 26 | 98 | 36 |
| Souiden et al | 2010 | African | 52 | 58 | 54 | 68 |
| Agalliu et al | 2006 | African | 22 | 9 | 8 | 7 |
| Li et al | 2008 | Asian | 87 | 121 | 134 | 96 |
| Yang et al | 2006 | Asian | 64 | 99 | 90 | 112 |
| Mittal et al | 2004 | Mixed | 48 | 55 | 82 | 35 |
| Thakur et al | 2011 | Mixed | 63 | 87 | 110 | 62 |
| Vijayalakshmi et al | 2005 | Mixed | 57 | 18 | 85 | 15 |
| Safarinejad et al | 2011 | Mixed | 96 | 72 | 242 | 94 |
| Komiya et al | 2005 | Asian | 93 | 93 | 131 | 157 |
| Murata et al | 1998 | Asian | 58 | 57 | 117 | 87 |
| Nakazato et al | 2003 | Asian | 43 | 38 | 52 | 53 |
| Kwon et al | 2011 | Asian | 76 | 90 | 202 | 125 |
| Lai et al | 2005 | Asian | 39 | 57 | 66 | 55 |
| Ashtiani et al | 2011 | Mixed | 60 | 50 | 90 | 10 |
| Gsur et al | 2001 | Caucasian | 91 | 75 | 85 | 81 |
| Nam et al | 2003 | Caucasian | 248 | 235 | 282 | 266 |
| Autrup et al | 1999 | Caucasian | 62 | 91 | 134 | 154 |
| Steinbrecher et al | 2010 | Caucasian | 122 | 126 | 221 | 270 |
| Steinhoff et al | 2000 | Caucasian | 46 | 45 | 70 | 57 |
| Medeiros et al | 2004 | Caucasian | 65 | 77 | 92 | 91 |
| Sivonova et al | 2009 | Caucasian | 60 | 69 | 98 | 130 |
| Aktas et al | 2004 | Caucasian | 81 | 19 | 93 | 14 |
| Silig et al | 2006 | Caucasian | 54 | 98 | 117 | 52 |
| Kote-Jarai et al | 2001 | Caucasian | 120 | 153 | 135 | 135 |
| Agalliu et al | 2006 | Caucasian | 248 | 311 | 274 | 278 |
| Beer et al | 2002 | Caucasian | 50 | 61 | 74 | 73 |
| Joseph et al | 2004 | Caucasian | 81 | 97 | 123 | 142 |
| Kelada et al | 2000 | Caucasian | 137 | 120 | 223 | 195 |
| Kidd et al | 2003 | Caucasian | 116 | 84 | 88 | 100 |
| Lavender et al | 2009 | Caucasian | 141 | 47 | 441 | 137 |
| Nock et al | 2006 | Caucasian | 222 | 216 | 241 | 238 |
| Kumar et al | 2011 | Mixed | 23 | 34 | 31 | 15 |
| Lima et al | 2008 | Mixed | 56 | 69 | 47 | 53 |
| Rodrigues et al | 2010 | Mixed | 83 | 71 | 68 | 86 |
| Caceres et al | 2005 | Mixed | 65 | 37 | 102 | 30 |
