# Supplementary material for: Association of GSTM1 Null Allele with Prostate Cancer Risk: Evidence from 36 Case-Control Studies
Source: PLoS One. 2012 Oct 10;7(10):e46982. doi: 10.1371/journal.pone.0046982 (PMC3468624; doi:10.1371/journal.pone.0046982)
Supplement: Table S2 — Main result of pooled ORs in the meta-analysis. (DOC) [file pone.0046982.s002.doc]

Table S2 Main result of pooled ORs in the meta-analysis.

|  | Null versus Present | | | |
| --- | --- | --- | --- | --- |
|  | N^a^ | OR(95%CI) | *P*^b^ | *P_heterogeneity_* |
| Total | 36 | 1.28(1.11-1.48) | 0.001 | < 0.01 |
| Ethnicity |  |  |  |  |
| Caucasian | 17 | 1.12(0.96-1.31) | 0.16 | < 0.01 |
| Asian | 7 | 1.35(1.03-1.78) | 0.03 | 0.01 |
| Smoking status |  |  |  |  |
| Non-smoking | 6 | 1.25(0.64-2.45) | 0.52 | < 0.01 |
| Smoking | 6 | 1.16(0.95-1.43) | 0.15 | 0.92 |

a, N, number of studies included in the meta-analysis.

b, *P* value of Z-test for OR test.
